# Prognostic value of cross-omics screening for kidney clear cell renal cancer survival

**DOI:** 10.1186/s13062-016-0170-1

**Published:** 2016-12-20

**Authors:** Slavica Dimitrieva, Ralph Schlapbach, Hubert Rehrauer

**Affiliations:** Functional Genomics Center Zurich, ETH Zurich and University of Zurich, Winterthurerstrasse 190, Zurich, 8057 Switzerland

## Abstract

**Background:**

Kidney renal clear cell carcinoma (KIRC) is a type of cancer that is resistant to chemotherapy and radiotherapy and has limited treatment possibilities. Large-scale molecular profiling of KIRC tumors offers a great potential to uncover the genetic and epigenetic changes underlying this disease and to improve the clinical management of KIRC patients. However, in practice the clinicians and researchers typically focus on single-platform molecular data or on a small set of genes. Using molecular and clinical data of over 500 patients, we have systematically studied which type of molecular data is the most informative in predicting the clinical outcome of KIRC patients, as a standalone platform and integrated with clinical data.

**Results:**

We applied different computational approaches to preselect on survival-predictive genomic markers and evaluated the usability of mRNA/miRNA/protein expression data, copy number variation (CNV) data and DNA methylation data in predicting survival of KIRC patients. Our analyses show that expression and methylation data have statistically significant predictive powers compared to a random guess, but do not perform better than predictions on clinical data alone. However, the integration of molecular data with clinical variables resulted in improved predictions. We present a set of survival associated genomic loci that could potentially be employed as clinically useful biomarkers.

**Conclusions:**

Our study evaluates the survival prediction of different large-scale molecular data of KIRC patients and describes the prognostic relevance of such data over clinical-variable-only models. It also demonstrates the survival prognostic importance of methylation alterations in KIRC tumors and points to the potential of epigenetic modulators in KIRC treatment.

**Reviewers:**

An extended abstract of this research paper was selected for the CAMDA Satellite Meeting to ISMB 2015 by the CAMDA Programme Committee. The full research paper then underwent one round of Open Peer Review under a responsible CAMDA Programme Committee member, Djork-Arné Clevert, PhD (Bayer AG, Germany). Open Peer Review was provided by Martin Otava, PhD (Janssen Pharmaceutica, Belgium) and Hendrik Luuk, PhD (The Centre for Disease Models and Biomedical Imaging, University of Tartu, Estonia). The Reviewer comments section shows the full reviews and author responses.

**Electronic supplementary material:**

The online version of this article (doi:10.1186/s13062-016-0170-1) contains supplementary material, which is available to authorized users.

## Background

Multi-omics datasets are now available for many cancers and provide a plethora of molecular details about the tumor tissues. The generation of these datasets has been driven by technological advancements that made genetic, epigenetic, transcriptomic and proteomic profiling possible. These data are informative for multiple aspects ranging from discovering of new markers for more accurate cancer diagnosis and prognosis, to development of new therapeutics and personalized treatments. With focus on kidney renal clear cell carcinoma (KIRC), as a response to one of the CAMDA 2015 challenges, we performed a systematic analysis of genome-wide molecular datasets to investigate underlying mechanisms of cancer progression.

Renal cell carcinoma is the most common neoplasm of the kidney and it accounts for approximately 95,000 deaths per year worldwide [1]. Early stage renal cell carcinoma is usually treated surgically and has an overall survival of 60–70%. However, late stage renal cell carcinoma has a poor prognosis with 5-year survival of less than 10% and it has limited therapeutic options. More than 30% of patients develop metastatic progression after therapeutic treatment. Among others, failure of currently known treatments can be attributed to cancer heterogeneity and an incomplete knowledge about the molecular determinants of cancer progression, which could be remedied by an appropriate omics screening of patients in the clinics.

In the last few years, extensive efforts have been made to incorporate diverse molecular information for better prognosis and treatment plans [2–4]. However, due to the rather high effort of large-scale molecular profiling, in practice clinicians are mainly focusing on a small number of selected genes or are using only single-platform genomic data. In this situation, we aimed to determine to what extent different molecular profiling data could be useful in clinical practice for cancer prognosis.

In this manuscript we present three computational strategies to preselect survival prognostic markers based on quantitative omics measurements and patient survival. Using these strategies we analyzed full multi-omics TCGA data [5] from more than 500 patients and identified genomic loci that are frequently altered in KIRC patients and are linked to patients survival. Then, for each molecular data type alone and in combination with each other and with clinical variables we evaluated the ability to predict patient survival.

## Methods

### Data

Clinical information of 533 patients (357 alive and 176 deceased) was obtained from the TCGA online database (http://tcga-data.nci.nih.gov, on October 22, 2015). Patient distribution by the TNM staging system was as follows: tumor stage I: 267, stage II: 57, stage III: 126 and stage IV: 86 patients.

Preprocessed molecular data were downloaded from the ICGC Data Portal (https://dcc.icgc.org), such that mRNA/miRNA/protein expression and somatic copy number variations (CNV) data were obtained from release 19, while DNA methylation data from release 18. Somatic mutation data were downloaded from the TCGA online database on October 22, 2015. For mRNA expression quantification we only used data coming from Illumina mRNA-seq experiments.

The samples that we analyzed come from two tissue types: primary tumor solid tissue and normal tissue adjacent to primary tumor.

### Data preprocessing

In CNV data analyses, protein-coding genes were mapped to genomic segments using the R package “GRanges” [6]. In the survival prediction analyses, we have considered only genes/probes whose expression, methylation or CNV levels were quantified in more than half of the patients. All statistical analyses were conducted in R version 3.2.0 [7].

### Identification of prognostic markers associated with overall patient survival

The patients were assigned into three equally sized sets: n_1_ = 178, n_2_ = 178 and n_3_ = 177. To make sure that no clear differences were observed in the three data sets in terms of survival time and vital status, we first sorted the patients based on their survival/follow-up time and then we distributed each of the consecutive patients to one of the sets. All computations were repeated in three rounds, such that at each round two sets were used as a training cohort, while the remaining set was used as a test cohort. This cross-validation technique assures that all patients were seen once in the test cohort and minimizes the possible bias in the results arising from patient stratification. Below we present the computational steps performed at each round.

On each omics data (mRNA/miRNA/protein expression, CNV and DNA methylation) we applied four different approaches to identify survival associated genomic loci:

- “Extreme score stratification approach”: The training cohort, which was composed from 2/3 of the patients, was randomly divided into two sets. For each omics data and for each gene/probe, we identified patients that have “extremely” high or “extremely” low quantitative molecular levels (expression/methylation/structural variation, respectively) in the first set. Next, we compared the overall survival of the patients that have “extremely” high molecular levels to the survival of the patients that have “extremely” low molecular levels using log-rank statistical test. If the survival was significantly different (*p*-value < 0.05), we tested if the same holds in the second set of patients. If the gene/probe was validated as predictive in the second set as well (*p*-value of log-rank test < 0.05), it was selected as a predictive marker. This procedure of randomly splitting the training cohort into two sets was repeated 100 times, and for each genomic loci the frequency of being selected as a predictive marker was counted. Quantitative molecular values were transformed to Z-scores by subtracting the average, then dividing by standard deviation of the respective molecular values in the tumor samples. The stratification of the patients into groups that have “extremely” high or “extremely” low quantitative molecular levels was done based on the Z-scores: Z-scores > 1 were noted as extremely high, Z-scores < −1 were noted as extremely low. We required that each stratified patient group contains at least 10 patients, to ensure that the selected predictive markers are informative for substantial set of patients and avoid selecting predictors that appear as relevant for individual patients only.
- “Mean score stratification approach”: Here for each omics data and for each gene/probe, we compared the survival of the patients that have higher than average quantitative molecular levels to the survival of patients that have lower than average quantitative levels. In this respect, we applied the same procedure as in the “extreme score stratification” approach, but we used a threshold of Z-score = 0 to stratify the patients (Z-score < 0 corresponds to lower than average; Z-score > 0 corresponds to higher than average).
- “Extreme survival stratification approach”: The training cohort was randomly divided into two sets. In the first set, we identified two groups of patients: the ones that died within the first year of diagnosis and the ones that lived longer than 5 years. Then for each omics data and for each gene/probe, we tested if there are significant differences in the quantitative molecular levels between the two groups of patients using *t*-test (*p*-value for significance < 0.05). If significant differences in the molecular levels were observed, the same procedure was applied on the second set. If significant differences were observed in the second set as well, the respective gene/probe was selected as a potential marker. For each set, we required to have at least 10 patients in the “short surviving” group, and at least 10 patients in the “long surviving” group. The procedure of randomly splitting the training cohort into two sets was repeated 100 times, and for each gene/loci the frequency of being selected as a predictive marker was counted, similarly as above.
  For each of the approaches we selected the top 10 most frequently selected genes/probes as survival predictive markers.
- “Combined approach”: in this approach we simply used the union of all the potential markers selected based on the above three approaches as survival predictive markers.

### Selection of predictive models

For each omics platform we used all possible combinations of predictive markers identified with each of the above-described approaches to build Cox regression models [8] on the training cohort. In the first three approaches, we built models with different ranks containing 1 to 10 selected predictive markers. For each rank (1 to 10) under each approach we selected the model that performs the best on the training cohort. Then we tested the selected models on the test cohort and reported their performance. In the “combined approach”, since the set of survival predictive markers consists of all markers selected under the other approaches, the number of possible predictive markers can range up to 30. In such case, examining all possible combinations of 10 selected markers is computationally very expensive; therefore in this approach we built models with ranks up to six (see Fig. 3).

The model performance on the train and test cohorts was measured via the concordance index (C-index) [9, 10]. The C-index is a nonparametric measure that quantifies the discriminatory power of predictive models. It is defined as the fraction of pairs of patients where the predicted survival times are correctly ordered among all pairs that can actually be ordered. A C-index of one indicates perfect prediction accuracy, while a C-index of 0.5 corresponds to a random guess.

In the integrative data analyses we used the union of all predictive markers from the different omics data to built multi-omics predictive models. The inclusion of a predictive marker into the model was assessed through a backward model selection procedure based on Akaike information criterion (AIC) [11] combined with a Cox regression. The computations were performed with the function *stepAIC* from the R package “MASS” [12], starting from an initial model that includes all predictive markers. The model that gives minimal AIC on the train data was evaluated on the test data. All computations were repeated in three cross-validation rounds.

## Results

### Identification of molecular signatures associated with overall patient survival in kidney renal clear cell carcinoma

To identify molecular signatures linked to patient survival in Kidney Renal Clear Cell Carcinoma (KIRC) we used clinical and multi-omics data from 533 patients. The patients were assigned into three equally sized sets. Two sets comprised the training cohort that was used to define prognostic signatures from each molecular platform and to define prognostic models, while the third set was used for testing the prognostic performance.

**Fig. 1:**
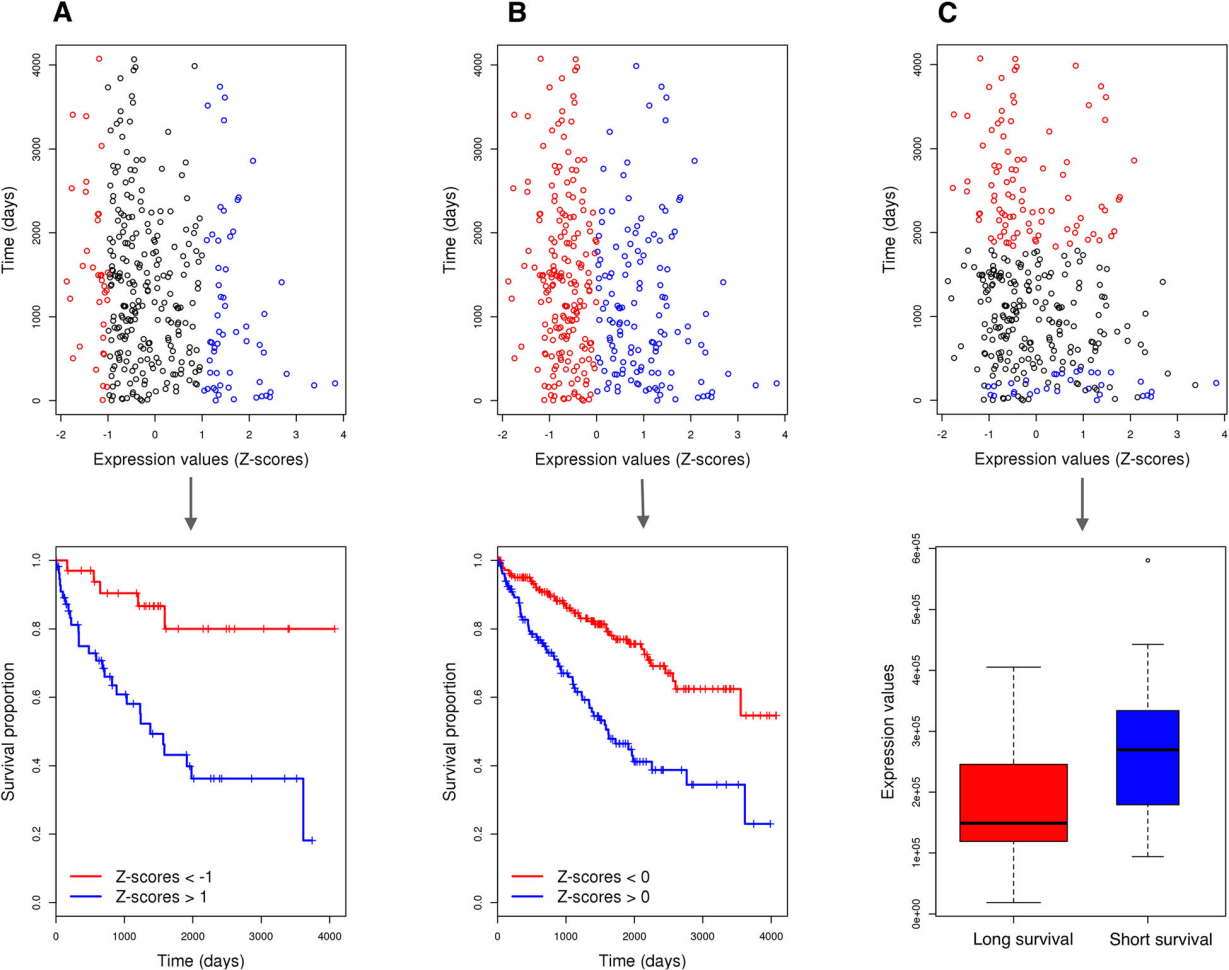
Feature selection process using three different approaches illustrated for the miRNA hsa-mir-21 in the KIRC cohort. **a** “Extreme score stratification approach”, where we compare the differences in the survival between “extremely” high expression values (Z-scores > 1, shown in *blue*) and “extremely” low expression values (Z-scores < −1, shown in *red*). **b** “Mean score stratification approach”, where we compare the differences in the survival between higher than average expression values (Z-scores > 0, shown in *blue*) and lower than average expression values (Z-scores < 0, shown in *blue*). **c** “Extreme survival stratification approach”, where we search for significant expression differences between patients that died within the first year of diagnosis (shown in *blue*), and patients that lived longer than 5 years (shown in *red*)

To assess which omics data has the best survival prediction power we applied four different approaches for selecting prognostic molecular signatures. First, we asked whether “extremely” low or high levels of a given quantitative molecular marker (miRNA/mRNA/protein expression, CNV or DNA methylation) had a significant correlation with patient overall survival (see Fig. 1a). Based on this “extreme score stratification approach” we selected the top loci from each omics data whose extreme measured values were statistically linked to patient overall survival. A variation of this approach has shown very good performance for detecting survival-associated miRNA signatures in KIRC [13].

In another approach, which we call “mean score stratification approach”, for each omics entity we compared the overall survival of the patient group characterized by measured levels lower than the average to the survival of the patient group with measured levels higher than the average (see Fig. 1b).

In our next approach, we only considered patients that died within the first year of diagnosis and patients that survived more than 5 years, and for each omics entity (miRNA/mRNA/protein expression, CNV or DNA methylation) we sought to determine if there are significant differences in the measured levels between the two groups of patients. This approach we call “extreme survival stratification approach” (see Fig. 1c).

To prioritize the loci that are most predictive for patient survival in all three approaches, resampling without replacement was performed on the training data (see Methods). Based on the selected survival predictive loci, we built multivariate Cox regression models [8] using data from the respective molecular platforms. For each particular approach and each omics data, the model that showed the best performance on the training dataset was selected for performance evaluation on the test dataset. The accuracy of the prognosis methods was assessed through the concordance index. [9, 10]

Last, we used a combination of the above three approaches, which we call “combined approach”, where a union of all the loci comprising selected molecular signatures based on the above three approaches was used to built new multivariate Cox regression models for each molecular platform.

### Performance of “extreme score stratification”, “mean score stratification” and “extreme survival stratification” methods on different omics data validated on the test KIRC cohort

**Fig. 2:**
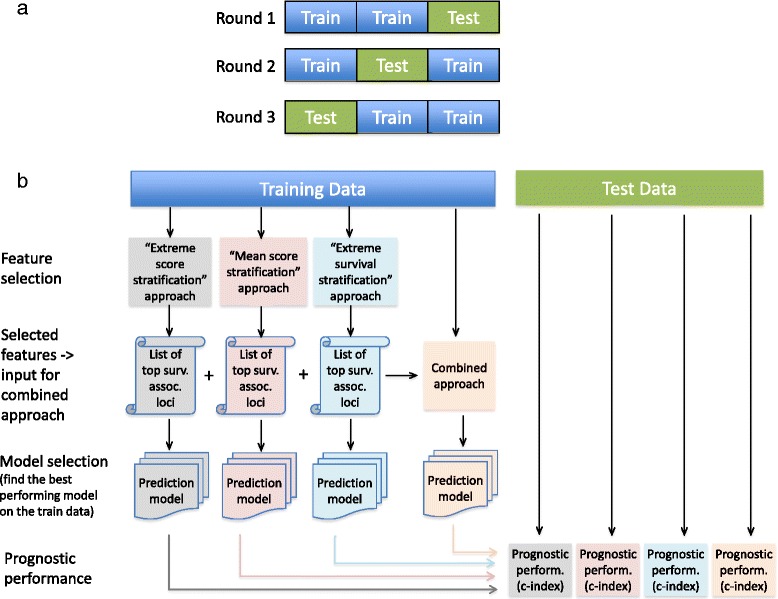
Flowchart of the analyses. **a** 3-fold cross validation procedure: the complete set of patients was distributed into three equally sized sets, and each time two sets were used as a training data, while the remaining set was used as a test data. **b** Computational steps performed at each cross-validation round on the training and test datasets

The performance of the predictive models selected on the training cohort was measured on the test cohort, which has not been seen during the feature selection and model selection steps. With the “extreme score stratification” and “extreme survival stratification” approaches, the feature selection procedure relies on the patients that have “extreme” values (omics measurements or survival times respectively). This could lead to a bias depending on the distribution of the patients with “extreme” measurements in the training and test cohorts. To eliminate any potential impact of the patients distribution into train and test cohorts on the feature selection and model selection steps, we performed 3-fold cross validation. In this respect, the KIRC patients cohort was divided into three equally sized sets, and the feature and model selection computations were repeated three times, each time using two of the sets as training data. After each training procedure, the excluded set was used for performance evaluation of the selected model (see Fig. 2).

**Fig. 3:**
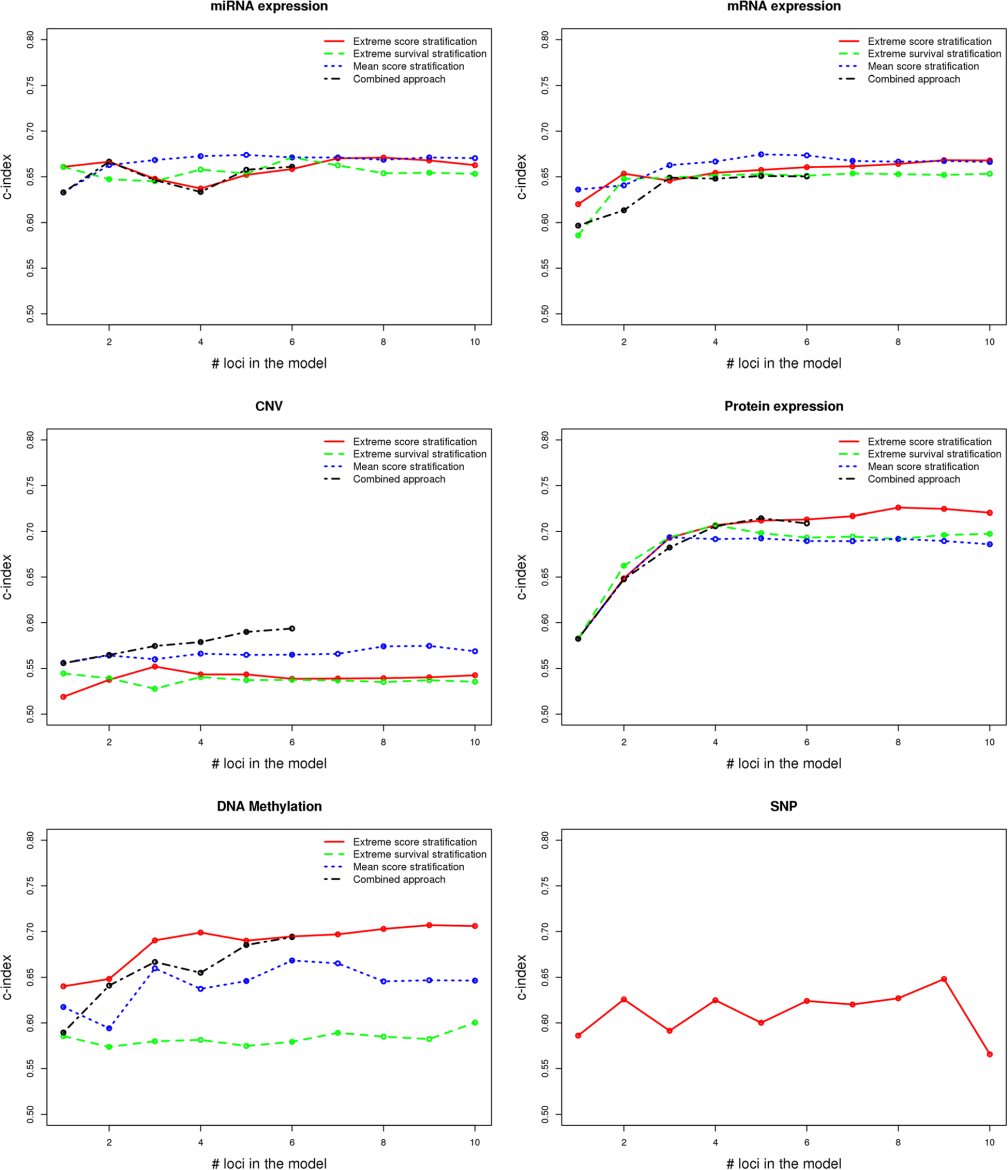
Performance of different feature selection approaches (“extreme score stratification”, “mean score stratification”, “extreme survival stratification” and combined approach) on different omics data on the KIRC cohort using 3-fold cross validation. The points at each plot show the average values across the three cross validation rounds. For clarity, the standard errors are omitted here, but are shown in Additional files 1 and 3

Each of the described approaches has led to prognostic models that have shown different performance for different omics data (Fig. 3). For thorough comparison of the respective approaches, we built and compared models with different complexities, such that the number of genomic loci included in the respective model ranges from 1 to 10.

Averaged across the three cross validation rounds, the “extreme score stratification” approach performs better than the other approaches for protein expression data and DNA methylation data (Fig. 3). The “mean score stratification” approach on average performs better than the other approaches for mRNA and miRNA expression data. However, none of the approaches is statistically significantly better than the others (see Additional file 1). The combined approach is not superior to the other three approaches because it tends to overfit the data, meaning that it always performs the best on the training data, but frequently it has suboptimal performance on the test data, except for the CNV data where it is the best performing method. However, these trends can be different in individual computational rounds, meaning that the survival predictive performance of the different approaches applied on the individual omics platforms can depend on the way the data is stratified into training and test cohorts. Additional file 2 shows the performance of the feature selection approaches on different omics data when only one stratification of the patients into train and test cohort is performed. Note that for this particular patient stratification, a model based on DNA methylation data built using the combined approach with six genomic loci performs the best compared to all other models (C-index = 0.78). Additional file 3 shows the standard deviations of the best performing approaches for individual omics platforms.

The performance of the survival predictive models based on mRNA/miRNA/protein expression and methylation data is significantly better than a random guess, as the confidence intervals for predictive power are above 0.5 (see Fig. 3 and Additional file 1).

We also included somatic mutation (SNP) data into our analyses (see Fig. 3), however the above-described approaches were not directly applicable on these data. To identify which mutated genes are linked to patient survival, for each gene we split the patients into two groups: patients having a somatic mutation in that particular gene, and patients with no somatic mutation in that gene. If the difference in the survival between the two patient groups is significant (*p*-value of log rank test < 0.05), we included the corresponding gene in the multivariate Cox model. Again the feature selection and model training was done on the training cohort, while the model performance evaluation was done on the test cohort.

Additionally, we tested whether individual SNPs within genes are informative for patient survival, such that we compared the survival of patients having a particular somatic mutation with the survival of patients with no such somatic mutation. However, we could not identify any individual somatic mutation that is directly linked to patient survival in the KIRC cohort. For successful identification of such SNPs, if any, a larger set of patients is required.

Note that using miRNA and mRNA expression data, Cox regression models based on only two loci have already relatively good predictive performance; the performance slightly increases when more genomic loci are added to the model. Models built based on protein expression data require at least 3 or 4 loci to be included in the model in order to achieve good performance.

**Fig. 4:**
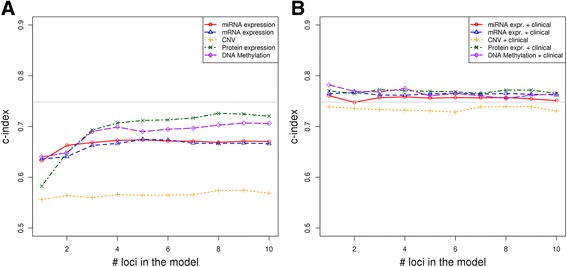
**a** Performance of predictive models built using individual omics data (miRNA/mRNA/protein expression, CNV segment means and DNA methylation). The *gray line* denotes the performance of the model based only on clinical variables (gender, age, tumor grade and tumor stage). **b** Performance of predictive models built using individual omics data (miRNA/mRNA/protein expression, CNV segment means and DNA methylation) integrated with clinical data (gender, age, tumor grade and tumor stage). The plots show only the results for the best predictive approach on each omics data, as shown on Fig. 3. The results were validated using 3-fold cross validation. For clarity, the standard errors are omitted here, but are shown in Additional file 6

We also constructed models based on clinical variables only, such that we included patient gender, age, tumor grade and tumor stage as clinical features. Notably, these models gave very good survival prediction (C-index = 0.748, st.dev = 0.024) and were superior to any of the predictive models built using molecular data only (see Fig. 4a). To examine whether omics data can provide additional prognostic power when used together with clinical variables, we built predictive models by integrating each type of molecular data with clinical variables (gender, age, tumor grade and tumor stage). These integrated models showed significantly improved predictive power compared to omics-data-only models (Fig. 4). Only the models based on expression and methylation data gave better survival prediction on average compared to clinical-variables-only models, however the prognostic gain was very limited (Fig. 4b). Interestingly, integrative models based on methylation and clinical data which rely on one or four methylation markers gave the best performance on average across the three cross validation rounds (C-indexes on test data are 0.78 and 0.77, respectively).

High-throughput molecular data from different platforms are not consistently available for all the patients. Tumor samples from only 402 patients have been characterized by all five molecular platforms that we analyzed; further 85 patients were characterized by four molecular platforms only, 28 patients by only three platforms; nine patients by only two platforms and for one patient molecular information was available from only one platform. For 12 patients, our collected data contained no information for any of the studied molecular profiles. The availability of molecular data per tissue type is summarized in Table 1. In our dataset mRNA expression data was the most commonly available data type across tumor samples. Protein expression data was available for only 123 proteins and not the entire proteome.

**Table 1:** Overview of high-throughput molecular data availability by tissue type in TCGA KIRC patients

Molecular platform	# patients with molecular profile in tumor tissue	# patients with molecular profile in normal adjacent tissue
miRNA expression	493	71
mRNA expression	518	72
Protein expression	454	0
CNV	511	510
DNA methylation	477	358

### Molecular biomarkers associated with overall patient survival

Rather than building predictive models for patient risk quantification, we aimed to provide insights into the molecular background of KIRC progression by identifying candidate biomarkers that are associated with patient survival. These candidate biomarkers could potentially act as drug design targets for improved personalized therapies. Table 2 lists candidate molecular biomarkers that were identified by at least two of the approaches with frequency of 100%. Interestingly, some of these candidate biomarkers were identified as survival predictive only by the “extreme stratification” approaches (the “extreme score stratification” and “extreme survival stratification”).

**Table 2:** Molecular biomarkers that were identified by at least 2 of the approaches with frequency of 100% in any of the three cross-validation rounds

Molecular type	Molecular biomarker	Extreme score stratific.	Mean score stratific.	Extreme survival stratific.	Survival prognosis association
miRNA	hsa-mir-10b	✓	✓	✓	High expression in better outcome
	hsa-mir-130a; hsa-mir-21	✓	✓	✓	High expression in worse outcome
	hsa-mir-190; hsa-mir-204; hsa- mir-676	✓		✓	High expression in better outcome
	hsa-let-7i	✓		✓	High expression in worse outcome
	hsa-mir-130b; hsa-mir-18a; hsa- mir-365-1; hsa-mir-223; hsa- mir-92b		✓	✓	High expression in worse outcome
	hsa-mir-3613	✓	✓		High expression in worse outcome
	hsa-mir-374b; hsa-mir-590	✓		✓	High expression in worse outcome
mRNA	ADH5; ARHGAP24; CLDN10; EHHADH; EIF4EBP2; FBXL5; GIPC2; IMPA2; MFSD4; SALL1; SORBS2; TPRG1L; LRBA; RBM47; RETSAT; RGNEF; SH3BGRL2	✓	✓	✓	High expression in better outcome
	AMOT; BBS1; CDC14B; EPHX2; FARS2; KCNJ15; PINK1; RAB3IP; STK32B; ZNF704;	✓	✓		High expression in better outcome
	ACADM; ALDH6A1; AMD1; ANK3; ATP11A; C5orf23; CCDC121; CLCN5; CPT2; CRYL1; CYFIP2; DDAH1; DMRTA1; FCHO2l; MAP7; MIA2; MOBKL2B; MRPS18B; NPR3; PANK1; PRKAA2; PRUNE2; SLC16A12; SLC27A2; SPATA18; TFEC; TMEM192; TMEM27; TMEM38B; TOX3; WDR31;		✓	✓	High expression in better outcome
	ACOX1; ALDH3A2; HLF; TIMP3; TMEM150C; UFSP2;	✓		✓	High expression in better outcome
Protein	AR; CTNNA1; CTNNB1; GAB2	✓	✓	✓	High expression in better outcome
	ACACA; CDKN1A; EA15; RAD51	✓	✓	✓	High expression in worse outcome
	ERRFI1; IGF1R; MAPK1 MAPK3; SHC1	✓	✓		High expression in better outcome
	EEF2	✓	✓		High expression in worse outcome
	TSC2		✓	✓	High expression in better outcome
	IGFBP2; VASP		✓	✓	High expression in worse outcome
DNA methylation probes	cg03032025 (CPEB4)		✓	✓	High methylation in worse outcome
	cg14827391 (NXN) cg15743907 (PDE4DIP) cg16419354 (FAM163A) cg24332577 (SALL4)	✓	✓		High methylation in worse outcome

Micro RNAs are actively involved in KIRC pathogenesis and several of them have been extensively studied for their role in cancer initiation and progression [14–19]. Our results show that high expression of mir-21, an established “oncomir” associated with a wide variety of cancers [16], strongly correlates with worse outcome prediction (see also Fig. 1). This miRNA has the highest prediction accuracy of all miRNAs and it was selected in the single-loci miRNA models as the most predictive. While high expression of mir-10b is associated with worse outcomes in some types of cancer [17], high expression of mir-10b is associated with better outcomes in KIRC patients. We identified several protein coding genes as informative for patient survival by the three approaches with frequency of 100%. Higher expression of these genes is linked to better prognosis (Table 2). Several known oncogenes are on our list of most frequently selected predictive protein-coding genes (SORBS2, LRBA, SH3BGRL2, AMOT, ACADM, HLF, TIMP3). Our list of survival-associated genes compiled using protein expression data was dominated by oncogenes: GAB2, ERRFI1, CTNNA1, CTNNB1, IGF1R, AR, SHC1, CDKN1A, IGFBP2 and TSC2. The monitoring of the expression of these genes/proteins might be useful in the clinical practice.

Using CNV data, no genes were identified as predictive jointly by two approaches. Applying our approaches on CNV data we could identify survival informative genes, however during the resampling process they were typically selected with lower frequencies (<60%). The top 10 selected genes based on CNV data by different approaches never overlapped and gave the worse predictive performance compared to the other omics data.

**Fig. 5:**
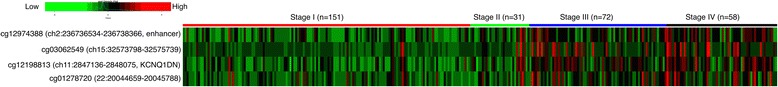
Stage specific methylation changes. Higher methylation levels (shown in *red*) are observed in stage III and stage IV patients, while lower methylation levels (in *green*) are observed in stage I and stage II patients. “cgX” denotes the identifier of the plotted methylation probe

DNA methylation is a common epigenetic alteration that has been reported in many cancers [20–22]. Recent high-resolution methylome study of KIRC patients demonstrated that many kidney specific enhancers are targeted by aberrant hypermethylation and are prognostic for overall survival [23]. In line with these results we have also identified many loci whose methylation status is informative for overall survival (Table 2 lists only a few of them, a longer list is given in Additional file 4). Few of the prognostic methylation markers that we identified seem to be correlated with the tumor stage: later stage tumors tend to have increased methylation at these loci (Fig. 5). In general, in the majority of the genomic loci whose methylation status is associated with overall survival we observed hypermethylation across tumor samples (see Additional file 5). This hypermethylation was generally linked to poor prognosis. The three methylation markers selected by the single loci models in the three rounds are: cg26813907 (C19orf21), cg16419354 (FAM163A) and cg02812891 (ECEL1P2). These three markers were included in the higher rank models in combination with other methylation markers.

The gene VHL, the most frequently mutated gene in KIRC tumors [2], was not informative for patient survival. Only mutations in BAP1 and TP53 were selected as informative for a subset of patients: these genes appeared on the list of survival associated loci, but with very low frequency of being selected during the resampling process (<10%). A recent study has shown that somatic mutations within BAP1 are related to tumor progression, but they do not define a category of patients with a worse outcome [13].

**Fig. 6:**
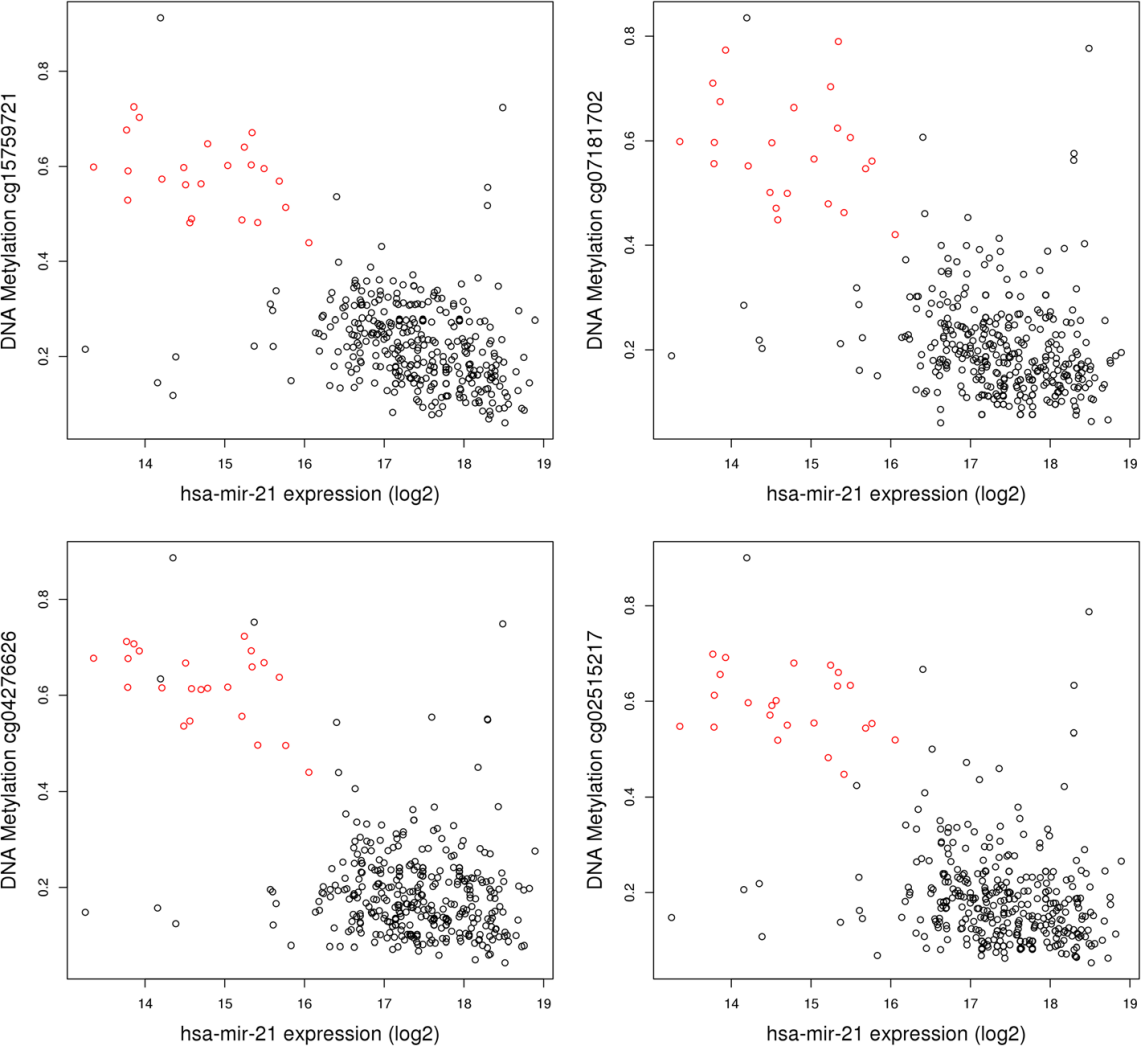
Interconnection between DNA methylation levels and RNA abundance illustrated for mir-21 in normal (*red points*) and tumor samples (*black points*). KIRC tumor samples are characterized by lower methylation levels and increased mir-21 expression

Since the abundance of mir-21 is highly predictive for survival as a standalone marker, we investigated whether the high expression of mir-21 in KIRC tumor samples is due to epigenetic changes in tumors or DNA sequence alteration. Our analyses showed that the high mir-21 abundance in tumor samples is likely due to DNA methylation changes in tumors. As Fig. 6 shows, normal tissues have higher methylation along the mir-21 gene, compared to tumor tissues. In tumor tissues, the methylation in these loci is altered, which likely results in increase of mir-21 expression.

### Integrative data analyses

**Fig. 7:**
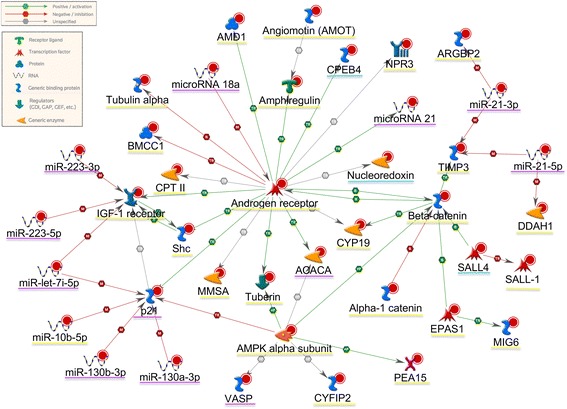
Interactions between some of the genes/proteins selected as survival predictive by our analysis. The shape of the nodes in this network corresponds to their biological function (see the legends on topleft). The genes/proteins that are underlined with purple are negatively associated with clinical outcome (i.e. higher expression is linked to poor survival); the ones underlined with yellow are positively associated with outcome (higher expression is linked to better survival). Higher methylation in genes underlined in blue is associated with worse outcome. This interaction network has been generated using MetaCore bioinformatics software version 6.26 build 68498 from Thomson Reuters https://portal.genego.com [41]

For understanding the complex biological processes that lead to cancer initiation and progression and extracting maximal biological insights from molecular data, the integration of diverse omics data is of central importance. It is crucial to know not only which genes are activated/suppressed in cancers, but also what are the interactions between these genes. In this respect, we searched for causal regulatory interactions between the genes selected as survival predictive markers from our study, confining the search to the genes selected jointly by at least two approaches (as presented in Table 2). Figure 7 shows a regulatory network between several survival-associated genes detected by our analyses. Central to this network is the androgen receptor (AR), a steroid-hormone activated transcription factor. In accordance with [2], our analyses have shown that higher expression of AR protein is associated with better outcome in KIRC. However, the role of AR in KIRC progression is not clear, as other studies have found negative correlation between AR expression and tumor stage [24]. The expression of AR is inhibited by miRNA-18a in prostate cancer [25], and our analyses show that lower expression of miRNA-18a is linked to better survival prognosis. AR transcriptionally regulates several other genes (see Fig. 7), among which is the IGF-1 receptor (IGF1R). IGF1R is a member of insulin receptor family and it has been shown that in prostate and breast cancer cells AR binds to IGF1R promoter and thus increases IGF1R expression [26, 27]. The expression of IGF1R is inhibited by miRNA-223 [28] and miRNA-let-7i [29] that negatively associate with KIRC survival. However, another study has shown that VHL inactivation in KIRC cells likely leads to IGF1R upregulation and this contributes to renal tumorigenesis and it is associated to worse outcome [30]. In contrast to this, but in line with [2] we observed positive correlation between IGF1R protein expression and KIRC outcome. There are also discrepancies concerning the impact of catenins, a family of cytoplasmic proteins, on KIRC initiation and progression. In prostate and bladder cancer decreased expression of β-catenin, E-cadherin, and α-catenin was correlated with poor survival [31, 32]. Our analyses on large cohort of KIRC patients have confirmed this survival association for α- and β-catenins (see also [2]). However, other studies on KIRC have found the opposite [33, 34]. More detailed investigations about the molecular function of these proteins in KIRC tumors need to be performed.

Finally, we integrated the selected prediction signatures from the different omics data together to build multi-omics survival prediction models (see Methods). However, the prognostic performance of this multi-omics prediction model has not improved significantly compared to the models from individual omics platforms (the C-index averaged across the three cross validation rounds was 0.708).

## Discussion

In this work we systematically evaluated patient survival prediction based on large-scale molecular data in ~500 KIRC patients from the TCGA database. We presented different computational approaches to identify survival associated genomic loci and applied them on the different molecular platforms to determine which omics data as a standalone platform give the best prediction for KIRC patient survival. Compared to previous studies, our analyses better support tumor heterogeneity across patients, since they were performed using different patient stratifications methods: we stratified the patients based on their quantitative molecular values, but also based on their survival times. Additionally, to make sure that our results are robust to patient distribution in test and training cohorts, all analyses were performed in three rounds, using 3-fold cross validation, so that each patient is seen once in the test cohort. In an earlier study with a similar goal, but using different approaches, Yuan et al. [35] established that molecular profiles from the TCGA can complement the survival prognosis based on clinical variables. Our analyses on KIRC patient cohort show that when molecular data alone are used for survival prediction, miRNA/mRNA/protein expression and methylation marks have statistically significant predictive powers compared to a random guess. We evaluated the prediction power of the molecular data using models relying on different number of predictive loci. In general, the predictive performance seems to saturate when more than six genomic loci were present in the models and did not improve significantly when more loci are added to the models. Interestingly, protein expression and DNA methylation data performed better than the other omics data on the KIRC cohort (C-index ≈ 0.7). Yet, in accordance to the findings in [35], clinical variables alone were the most informative for survival prediction in KIRC patients (C-index: 0.75). Importantly, integrative models accounting on both, molecular and clinical variables performed better than the clinical-variables-only model, however the gain in the prediction power was limited (maximal C-index ~0.78). The limitation of the molecular signatures to perfectly predict cancer survival supports the view that cancer is an extremely complex disease and it is heterogeneously defined within patients [4]. Additionally, the therapeutic treatments that patients receive after cancer diagnosis can have an impact on their survival and have to be accounted in the survival prediction models. However, the information about therapeutic treatments is frequently unavailable (in the TCGA KIRC cohort only 83 patients have information about administered drugs), and frequently the patients received a combination of drugs, which makes data inferences even more difficult.

Previous studies using TCGA data have shown that miRNA based signatures integrated with clinical variables yielded good prediction for KIRC patients [13, 35]. Our analyses confirm these results, but also shed light on the importance of protein expression and DNA methylation on alterations in KIRC tumorigenesis and progression. Our results show that simultaneous measurement of several differentially methylated genomic loci could result in good survival prediction, at least for a subset of patients. Most of the survival prognostic methylation markers that we identified are hypermethylations that occur in tumor tissues, but are absent in normal tissues, and some of them even correlate with the tumor stage. However, although previous studies have found that KIRC tumors frequently have alterations in genes with major roles in epigenetic regulation [2, 36–38], to our knowledge only a few studies on a small number of patients have explored the usability of DNA methylation markers as predictors of overall survival [39]. A recent study identified a set of DNA methylation biomarkers that can reliably distinguish tumor from benign adjacent tissue and can serve as clinically applicable biomarkers for early KIRC diagnosis [40]. The relationship of DNA hypermethylation to KIRC formation and progression is important to be considered in the light of epigenetic cancer therapies that can reprogram tumor cells toward a normal state.

## Conclusions

We evaluated the potential of different large-scale omics data in predicting the survival of patients with kidney renal clear cell carcinoma. Our results suggest that for estimating survival times of patients, in practice clinicians can rely on the clinical variables only. Models integrating both molecular and clinical variables performed statistically better than the clinical-variables-only model, but the gain in the prediction power was very limited. However, understanding the molecular changes is indispensable in disease related research. The identification of novel markers for diagnosis and survival prognosis can facilitate our understanding of the molecular biology of KIRC and can lead to identification of new points for therapeutic actions. Our analyses do not necessarily identify the KIRC causal changes; they rather identify molecular markers that are affected by causal changes and are associated with survival. They offer new prospects for further investigations of KIRC pathogenesis.

## Reviewers’ comments

### Reviewer’s report 1: Martin Otava, PhD, Janssen Pharmaceutica, Belgium

**Reviewer summary:**

The paper is well written and used methodology seems to be appropriate. The authors approach the multiple data sources with algorithm that is simple enough to follow it, but simultaneously well designed and cross-validated. Their interpretation of results is clear and added value of their research and possible limitations are nicely summarized. I consider the manuscript as very good example how to extract information from multiple high dimensional data sources and how to consequently communicate the results with scientific public.

Still, there were few details that should be clarified for the reader prior to acceptance of the manuscript. My comments regarding this matter are summarized below.

**Reviewer recommendations to authors:**

1. pg 1: You claim that “Our analyses show that expression and methylation data have statistically significant predictive powers compared to a random guess, but do not perform better than predictions on clinical data alone.”

However, I have not seen in the paper any formal statistical justification of this claim. I understand that it should be somehow based on the fact that confidence intervals for predictive power are all above 0.5, but it should be stated somewhere in manuscript explicitly.

Authors’ Response: *We would like to thank to the reviewer for all valuable comments. Our claim that expression and methylation data have statistically significant predictive powers compared to a random guess is indeed based on the fact that confidence intervals for predictive power are all above 0.5. This can be seen from the newly added Additional files* 1 *and* 6 *. We have added a text in the manuscript explicitly stating that.*

2. pg 3: In Section “Selection of predictive models”, you explain that you fit Cox models with 1–10 predictors. However, in Combined approach, you use union, so you can potentially end up with 30 predictors. What will you do in such a case? Please, elaborate on this in the respective section.

Authors’ Response: *In the “combined approach”, the number of possible predictive markers can ranges up to 30 (actually in our data it goes from 17 to 30). To fit a Cox model with 10 predictive markers for example, we need to examine all possible combinations of 10 selected markers (out of 30) and this is computationally very expensive. Because of that, in this approach we built models with ranks up to six (this can be seen on Fig.* 3 *). Regarding this, we have now added an explanation in the section “Selection of predictive models”.*

*As stated on page 6, the Cox models built using this approach tend to overfit the data, so we do not expect that their performance on the test data will be improved by adding more predictor variables in the models. Therefore, running highly expensive computations is not justified.*

3. pg 4: These page should be pruned significantly, because lot of information is redundant given thorough descrition in Methods section. The description of algorithm is not needed here (especially given that it is repeated again in caption of Fig. 1, which is actually very handy), present only the results here.

Authors’ Response: *We have shortened this section by removing the sentences were the algorithm description was redundant.*

4. pg 6: “The “mean score stratification” approach performs better than the other approaches for mRNA and miRNA expression data.”

Although you do not state anything about statistical significance here, it may give impression that there is some evidence for this conclusion further than means comparison. Looking at Additional file 3, I doubt that if you show all confidence intervals, any approach would be significantly different/better than other. It is all fine, but I would prefer to have it more clearly stated in manuscript that the differences are rather subtle.

Authors’ Response: *We have added all confidence intervals to the Additional file* 1 *. Indeed, none of the approaches is statistically significantly better than the others. We have added a sentence in the manuscript clearly stating that.*

5. pg 14: “Importantly, integrative models accounting on both, molecular and clinical variables performed better than the clinical-variables-only model, however the gain in the prediction power was limited (maximal C-index ~0.78).”

Based on this, would you actually suggest clinician in practise to use the molecular variables or to use clinical variables only and use molecular variables rather in disease related research than in everyday practice?

Authors’ Response: *Yes. Our results suggest that in practice the clinicians can rely on the clinical variables to give an estimate for the survival time of the patients. However, understanding the molecular changes is indispensable in disease related research and can lead to identification of new points for therapeutic actions. This is discussed in the* *Discussion* *section of the manuscript.*

6. Minor comments: pg 2: add reference to R in Data preprocessing section

Authors’ Response: *The reference is added (ref* [7]*).*

7. pg 2: “The patients were assigned into three equally sized sets: n1 = 178, n2 = 178 and n3 = 177, such that no bias in terms of survival time and vital status was observed in each of the sets.”

It is not clear, how the assignment was done. Were patients distributed randomly and then average survival time and vital status of groups were checked and no difference observed? Or have you distributed patients already in a way that survival time and vital status is similar in all three groups, based on some algorithm? The word “bias” does not seem appropriate here, I would rather state simply “no clear difference was observed among three sets, in terms of survival time and vital status”.

Authors’ Response: *We distributed the patients in a way that survival time and vital status is as similar as possible in all three groups. We proceeded such that we first ordered the patients based on their survival/follow-up time and then we distributed each of the consecutive patients to one of the three sets. This way there were no clear differences in the average survival time and vital status of the groups.*

*We have added an explanation about this in the manuscript (section “* *Identification of prognostic markers associated with overall patient survival* *”)*

8. pg 2: typo “survival- associated”

Authors’ Response: *The typo is corrected.*

9. pg 3: “We required that each stratified patient group contains at least 10 patients”

Please, add why you have chosen 10.

Authors’ Response: *We required that each stratified patient group contains at least 10 patients to make sure that our selected predictive markers are informative (common) for substantial set of patients, i.e. are as general as possible. This way we avoid selecting predictors that work only for 1–2 patients on the test data. But the choice of exactly 10 patients was somewhat arbitrary.*

*We added a text in the manuscript that describes this.*

10. pg 3: “For each of the approaches we selected the top 10 most frequently selected genes/probes as survival predictive markers.”

Please, separate visually from the text of approach 3, since it applies to all three approaches, no?

Authors’ Response: *Yes, it applies to all three approaches and we have separated it from the text of approach 3.*

11. pg. 14 typo in “Akaike”

Also, this should be mentioned in Methods, not here. Additionally, I would require more information on how “the forward model selection procedure combined with Cox regression” was done.

Authors’ Response: *The typo is corrected. We have added an explanation about this in the* *Methods* *section and added more information about the way we did the computations (last paragraph of the section “* *Selection of predictive models* *”). Additionally, we corrected one unintentional mistake: in our computations we actually used backward (instead of forward) model selection procedure.*

12. Throughout paper: make sure that there as spaces around inequalities “Z < 0” etc. It would improve readability

Authors’ Response: *We have corrected this.*

### Reviewer’s report 2: Hendrik Luuk, PhD, The Centre for Disease Models and Biomedical Imaging, University of Tartu, Estonia

**Reviewer summary:**

The paper is well written and it’s purpose is clear. The authors have tested four scenarios for identifying molecular features predictive of survival of 533 patients with kidney renal clear cell carcinoma. Model performance was estimated using 3-fold cross-validation and concordance index (C-index). The authors find that clinical variables alone were the most informative for survival prediction in KIRC patients. Some comments below.

**Reviewer recommendations to authors:**

1. Approximately, what fraction of measurements fell into the “extreme” group? For normally distributed, data one would expect around 15%, which sounds more like a “moderate” amount.

Authors’ Response: *We would like to thank this reviewer for the valuable comments on our manuscript. Generally, about 15% of samples fell into one “extreme” group. So under the “extreme score stratification” approach for each molecular value we consider roughly 30% of the samples in the calculations.*

2. How many iterations of the 3-fold cross-validation were performed (assuming each iteration contained patients randomly partitioned into three groups)? I’m asking this, because it would be nice to see error-bars in Figs. 3 and 4. Otherwise it is impossible to say whether there is a performance difference between the approaches. Supplementary figures appear to include error bars only for the best performing approach, which are not meaningful alone.

Authors’ Response: *In the feature selection procedure, we used resampling with replacement on the train data and performed 100 iterations. Based on the top selected features (predictors), in each cross validation round and for each model size we selected the best performing model on the train dataset and tested it on the validation dataset. So in each cross validation round we end up with one “final” model with a certain size (1–10 predictors) whose performance we evaluate. As we did 3-fold cross validation, the error bars are quite high. We have added new figures: Additional files* 1 *and* 6 *that correspond to Figs.* 3 *and* 4 *but include error bars. Additionally, we have added an explanation in the manuscript that none of the approaches is statistically significantly better than the others.*

3. I didn’t see a reference to the source of the regulatory network shown in Fig. 7.

Authors’ Response: *The regulatory network shown in Fig.* 7 *was generated using MetaCore bioinformatics software version 6.26 build 68498 from Thomson Reuters* *https://portal.genego.com* [41]. *This is now added to the caption of Fig.* 7 *.*

## Additional files

Additional file 1: Survival predictive performance on different omics data on the KIRC cohort using 3-fold cross validation. This is the same as Fig. 3, but with included standard errors for all approaches on each omics data. (PNG 496 kb)
Additional file 2: Performance of different feature selection approaches (“extreme score stratification”, “mean score stratification”, “extreme survival stratification” and combined approach) on different omics data on the KIRC cohort in the first cross validation round (i.e. when only one distribution of the patients into train and test cohorts is performed). (PNG 500 kb)
Additional file 3: Survival predictive performance on different omics data on the KIRC cohort using 3-fold cross validation. This is the same as Fig. 3, but for better clarity we included only the standard errors for the best performing approach on each omics data. (PNG 485 kb)
Additional file 4: List of DNA methylation loci that were identified as associated with KIRC patient survival by at least one of the feature selection approaches in any cross validation round. (XLS 39 kb)
Additional file 5: DNA methylation levels in tumor and normal samples. Only top survival predictive marks are shown. Higher methylation levels are shown in red, lower methylation levels are in green, middle methylation levels are in black. (PNG 2089 kb)
Additional file 6: A) Performance of predictive models built using individual omics data (miRNA/mRNA/protein expression, CNV segment means and DNA methylation). The gray line denotes the performance of the model based only on clinical variables (gender, age, tumor grade and tumor stage) B) Performance of predictive models built using individual omics data (miRNA/mRNA/protein expression, CNV segment means and DNA methylation) integrated with clinical data (gender, age, tumor grade and tumor stage). The plots show only the results for the best predictive approach on each omics data, as shown on Fig. 3. The results were validated using 3-fold cross validation. This is the same as Fig. 4, but with included standard errors. (PNG 274 kb)

## Abbreviations

AIC: Akaike information criterion
CNV: Somatic copy number variations
KIRC: Kidney renal clear cell carcinoma
TCGA: The Cancer Genome Atlas
