# Supplementary figures and images for: Prognostic value of cross-omics screening for kidney clear cell renal cancer survival
Source: Biol Direct. 2016 Dec 20;11:68. doi: 10.1186/s13062-016-0170-1 (PMC5168807; doi:10.1186/s13062-016-0170-1)

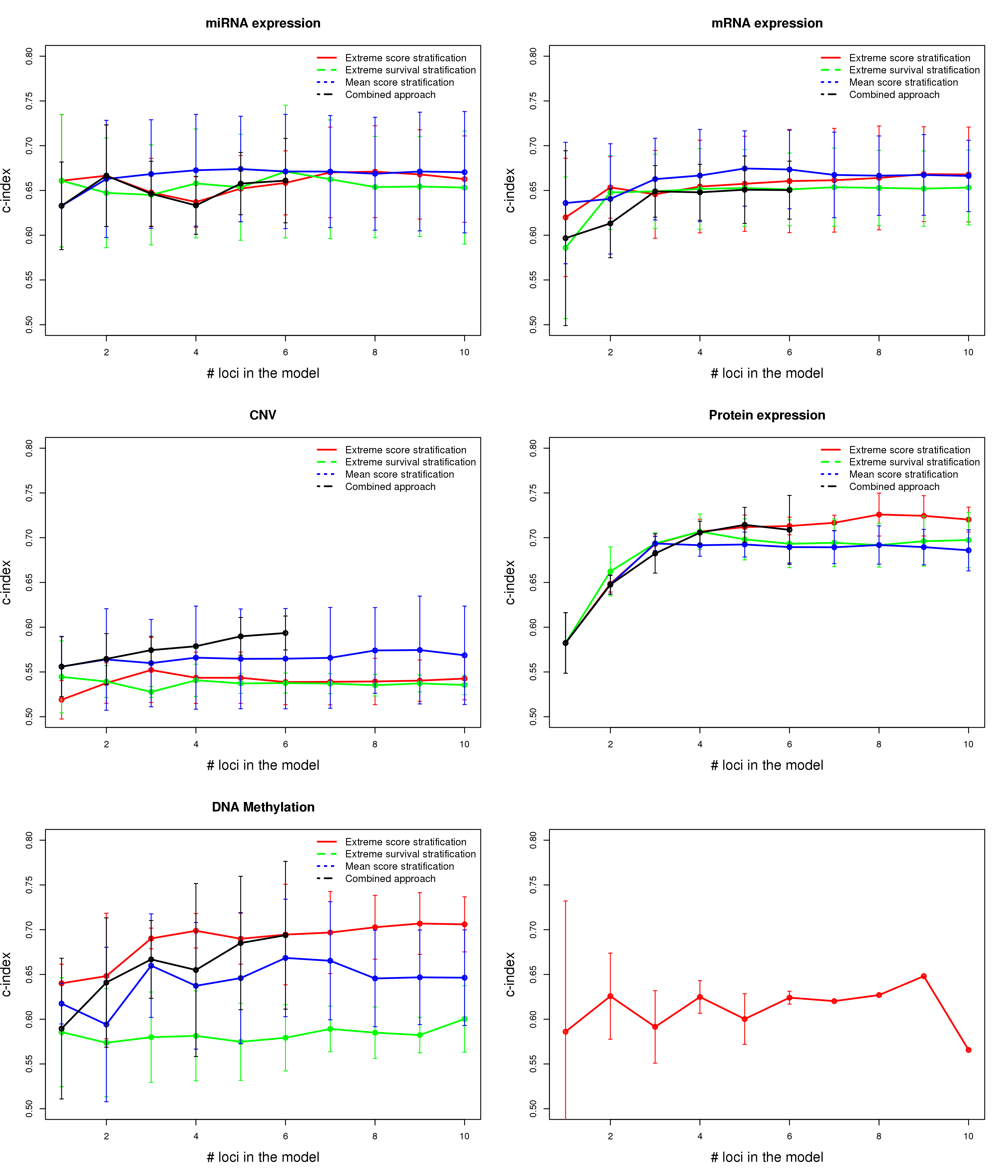

Supplement: Additional file 1: — Survival predictive performance on different omics data on the KIRC cohort using 3-fold cross validation. This is the same as Fig. 3, but with included standard errors for all approaches on each omics data. (PNG 496 kb) [file 13062_2016_170_MOESM1_ESM.png]

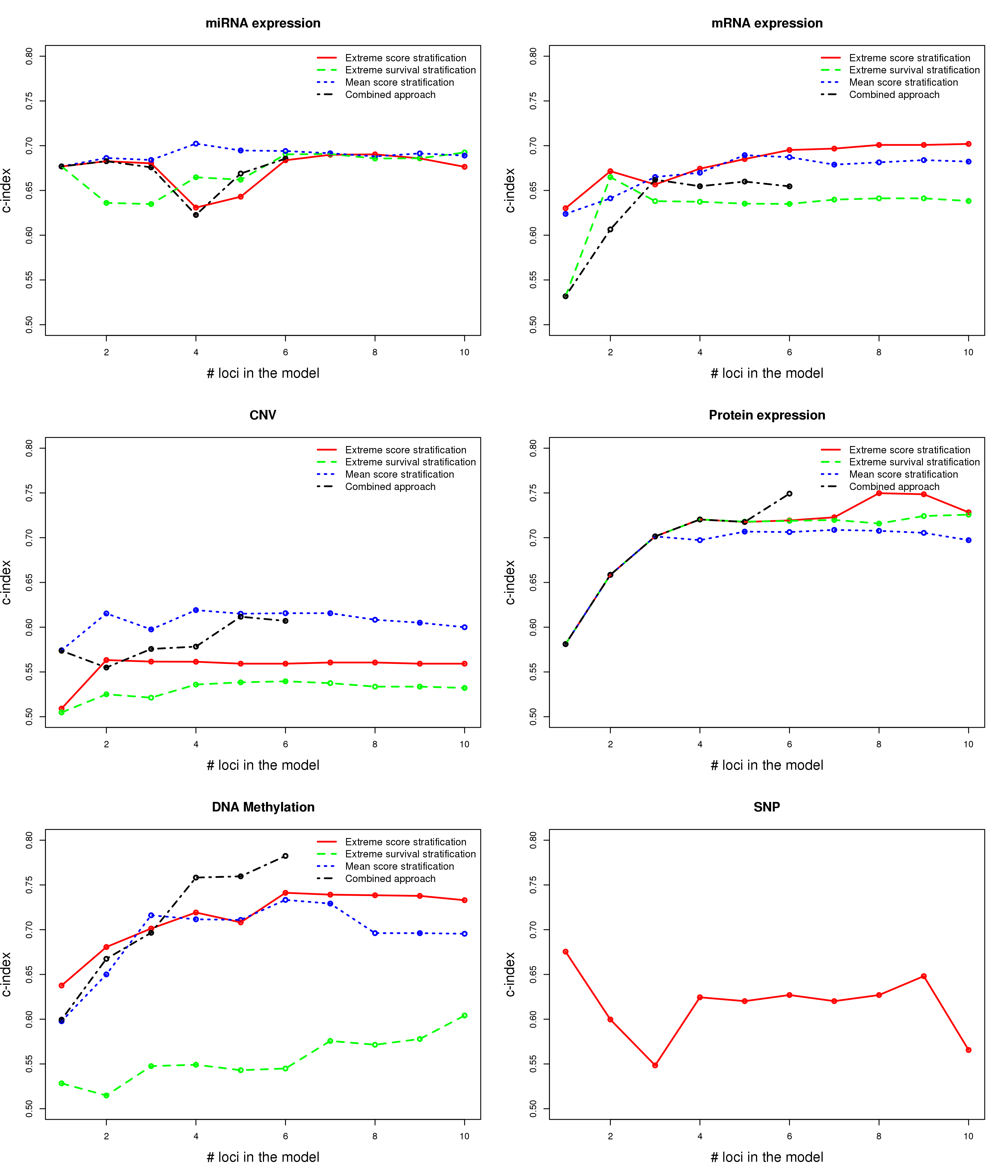

Supplement: Additional file 2: — Performance of different feature selection approaches (“extreme score stratification”, “mean score stratification”, “extreme survival stratification” and combined approach) on different omics data on the KIRC cohort in the first cross validation round (i.e. when only one distribution of the patients into train and test cohorts is performed). (PNG 500 kb) [file 13062_2016_170_MOESM2_ESM.png]

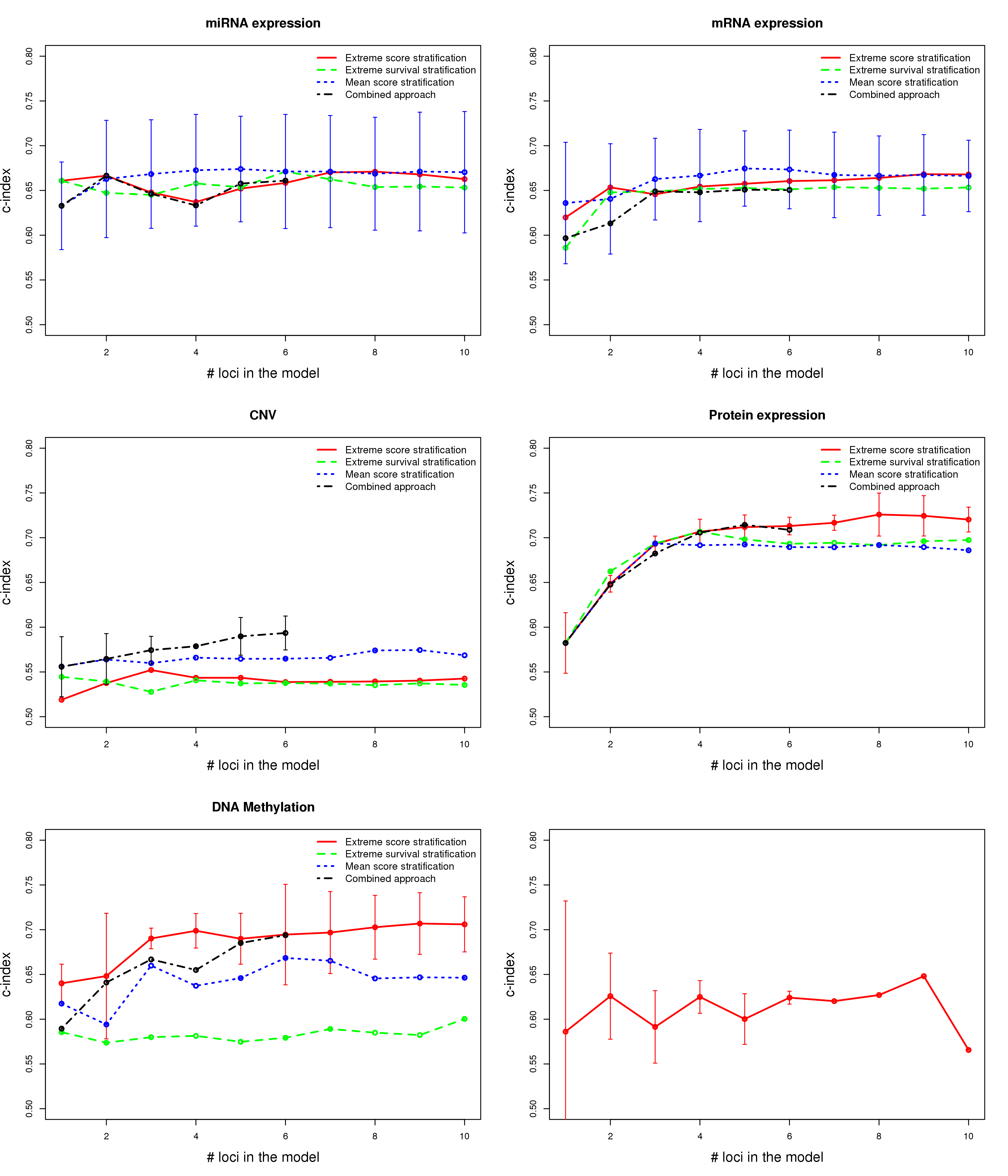

Supplement: Additional file 3: — Survival predictive performance on different omics data on the KIRC cohort using 3-fold cross validation. This is the same as Fig. 3, but for better clarity we included only the standard errors for the best performing approach on each omics data. (PNG 485 kb) [file 13062_2016_170_MOESM3_ESM.png]

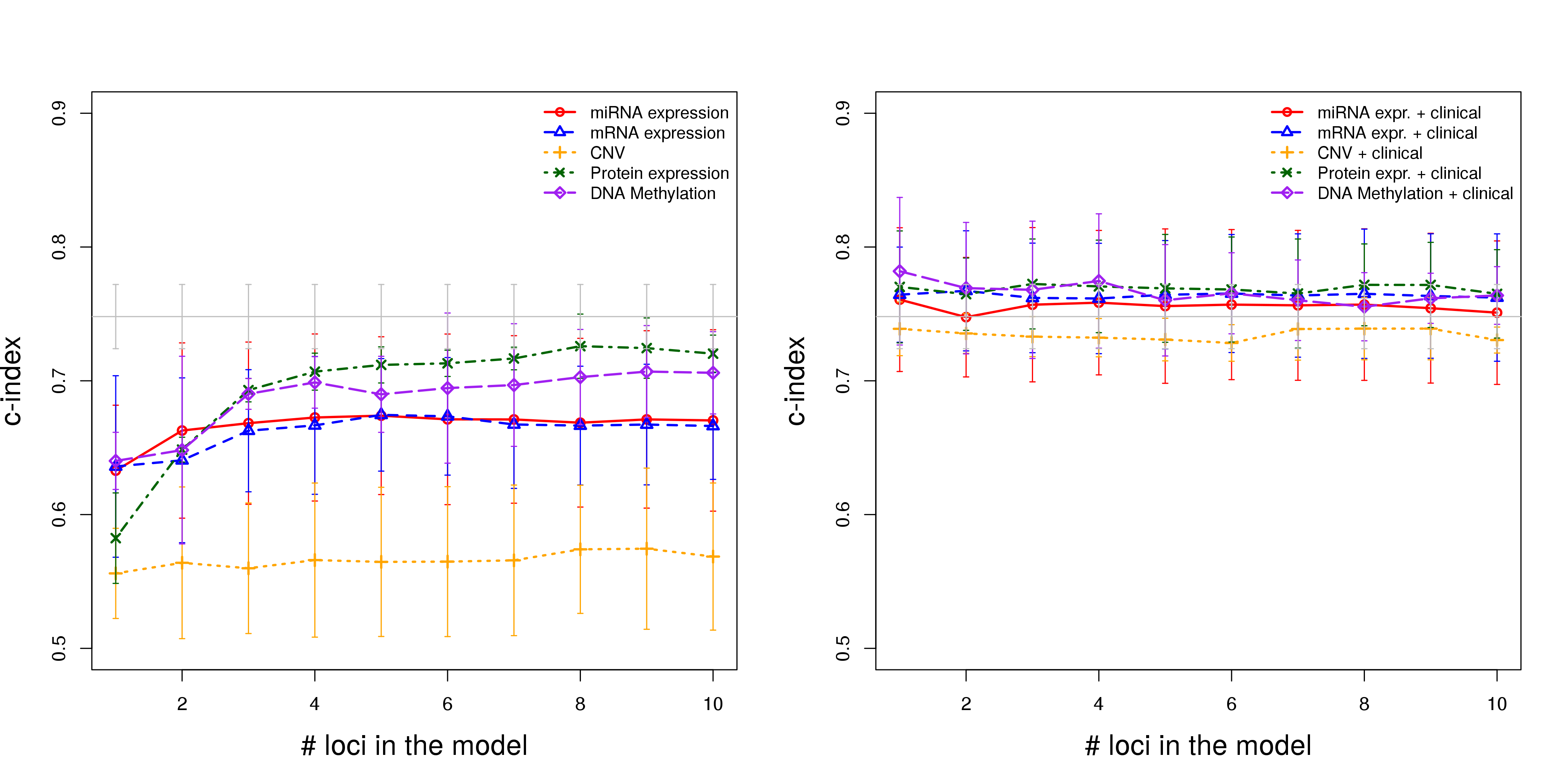

Supplement: Additional file 6: — A) Performance of predictive models built using individual omics data (miRNA/mRNA/protein expression, CNV segment means and DNA methylation). The gray line denotes the performance of the model based only on clinical variables (gender, age, tumor grade and tumor stage) B) Performance of predictive models built using individual omics data (miRNA/mRNA/protein expression, CNV segment means and DNA methylation) integrated with clinical data (gender, age, tumor grade and tumor stage). The plots show only the results for the best predictive approach on each omics data, as shown on Fig. 3. The results were validated using 3-fold cross validation. This is the same as Fig. 4, but with included standard errors. (PNG 274 kb) [file 13062_2016_170_MOESM6_ESM.png]
